# 

**Published:** 1847-03

**Authors:** 


### WESTERN JOURNAL

OF

### MEDICINE AND SURGERY:

EDITED BY

DANIEL DRAKE, M. D.

AND

LUNSFORD P. YANDELL, M. D.

PROFESSORS IN THE UNIVERSITY OF LOUISVILLE,

AND

THOMAS W. COLESCOTT, M. D.

NO. LXXXVII.—MARCH, 1847.

NEW SERIES.

VOL. VII.—NO. III.

LOUISVILLE, KY.

PUBLISHED MONTHLY, BY PRENTICE AND WEISSINGER,

PROPRIETORS AND PRINTERS.

1847.

